# Immunotherapy With Checkpoint Inhibitors in FGFR-Altered Urothelial Carcinoma

**DOI:** 10.1177/11795549221126252

**Published:** 2022-09-27

**Authors:** David J Benjamin, Nataliya Mar, Arash Rezazadeh Kalebasty

**Affiliations:** 1 Medical Oncology, Hoag Family Cancer Institute, Newport Beach, CA, USA; 2 Division of Hematology and Oncology, Department of Medicine, Chao Family Comprehensive Cancer Center, University of California, Irvine, Orange, CA, USA

**Keywords:** Urothelial carcinoma, bladder cancer, FGFR, erdafitinib, checkpoint inhibitor, immunotherapy

## Abstract

The treatment landscape of metastatic urothelial cancer (mUC) remained unchanged for over 30 years until the approval of immune checkpoint inhibitors (ICIs) in 2016. Since then, several ICIs have been approved for the treatment of mUC. In addition, recent molecular characterization of bladder cancer has revealed several subtypes, including those harboring fibroblast growth factor receptor (FGFR) mutations and fusion proteins. Erdafitinib, a pan-FGFR inhibitor, was approved for the treatment of metastatic/advanced UC in 2019. Some available evidence suggests ICI may have inferior response in advanced FGFR+ UC for unclear reasons, but may possibly be related to the tumor microenvironment. Several ongoing trials are evaluating erdafitinib in metastatic/advanced UC including the ongoing phase IB/II NORSE trial combining erdafitinib plus ICI, which may prove to offer a more robust and durable response in patients with FGFR+ metastatic/advanced UC.

## Introduction

Urothelial cancer (UC) is diagnosed in approximately 555 000 individuals yearly, and accounts for nearly 200 000 deaths worldwide.^1^ Risk factors for the development of UC include tobacco use, occupational exposures to aromatic amines and polycyclic aromatic hydrocarbons, and chronic inflammation from recurrent infections.^2^ Approximately 75% of cases of UC involve non-muscle-invasive bladder cancer (NMIBC), which is superficial to the muscularis propria. Muscle-invasive bladder cancer (MIBC) accounts for the remaining 25% cases of UC and has the potential to become metastatic. Advanced UC has spread to adjacent tissues and organs at the time of diagnosis.^2^ The treatment of NMIBC includes transurethral resection of bladder tumor (TURBT), intravesical Bacillus Calmette-Guérin (BCG), intravesical chemotherapy with either mitomycin or gemcitabine, and in cases of BCG-unresponsive NMIBC, either pembrolizumab or cystectomy.^3^ Transurethral resection of bladder tumor is an endoscopic procedure through the urethra that is performed by a urologist to obtain a biopsy and/or resection of a tumor in the bladder.^4^ Muscle-invasive bladder cancer is treated with cystectomy, with or without neoadjuvant chemotherapy, chemoradiotherapy, or radiotherapy. The landscape of treatment for metastatic urothelial cancer (mUC) remained unchanged for nearly 30 years until the approval of the immune checkpoint inhibitor (ICI) atezolizumab in 2016.^5^ Since then, several ICIs have been approved for the treatment of mUC including atezolizumab, nivolumab, durvalumab, avelumab, and pembrolizumab.^6^ However, of note, in February 2021, durvalumab was voluntarily withdrawn from the market for mUC by AstraZeneca, and in March 2021, Roche voluntarily withdrew atezolizumab for the treatment of previously platinum-treated mUC.^7^ Despite advancements in the available therapeutic options for mUC, patients who progress on first-line treatment have historically had poor outcomes.^8,9^

## Subtypes of UC

According to expression analysis from The Cancer Genome Atlas (TCGA), UC can be classified into 4 subtypes, including luminal cluster I, luminal cluster II, basal cluster III, and basal cluster IV.^10^ The TCGA analysis demonstrated that luminal cluster I subtype of UC consists of 30% to 35% of UC, is of papillary histology, and commonly has FGFR3 mutations. As such, treatment with an fibroblast growth factor receptor (FGFR) inhibitor may be particularly appropriate in these patients.^11^ In addition, luminal cluster II subtype of UC consists of 30% to 35% of UC, and has high signatures of human epidermal growth factor receptor-2 (HER2) and estrogen receptor signaling. Approximately 20% to 25% of UC cases consist of basal cluster III subtype, which has similar expression patterns as basal-like breast cancer and squamous cell cancers of the head, neck, and lung. Finally, the remaining 10% to 15% of UC is made up of basal cluster IV, which is similar to cluster III except that this subtype contains features of surrounding stroma and muscle.

More recently in 2020, the Bladder Cancer Taxonomy Group proposed an international consensus on MIBC molecular subtypes based off 1750 MIBC transcriptomic profiles from 16 published datasets and 2 additional cohorts.^12^ The study identified 6 molecular classes of MIBC including luminal papillary (24%), luminal non-specified (8%), luminal unstable (15%), stroma-rich (15%), basal/squamous (35%), and neuroendocrine-like (3%). Luminal papillary tumors were found to have high rates of FGFR3 mutations and translocations, suggesting that this molecular class may be responsive to treatment with FGFR inhibitors. Basal/squamous tumors were found to express high levels of epidermal growth factor receptor (EGFR), and may be sensitive to EGFR inhibitors. In addition, basal/squamous tumors express immune checkpoint markers and could potentially be more responsive to ICI. Table 1 provides a summary of the proposed molecular subtypes, with clinically relevant FGFR alterations highlighted in bold, as presented by TCGA and the Bladder Cancer Taxonomy Group.

**Table 1.** Subtypes of UC based off expression analysis.

Expression analysis	Subtype	Percentage of UC	Expression patterns
The Cancer Genome Atlas (TCGA)	Luminal cluster I	30%-35%	**Commonly has FGFR3 mutations**
	Luminal cluster II	30%-35%	High signatures of HER2 and estrogen receptor signaling
	Basal cluster III	20%-25%	Similar to basal-like breast cancer and squamous cell cancers of head and neck, and lung
	Basal cluster IV	10%-15%	Similar to cluster III
Bladder Cancer Taxonomy Group	Luminal papillary	24%	**High rates of FGFR3 mutations and translocations**
	Luminal non-specified	8%	No specific or potentially actionable expression signatures
	Luminal unstable	15%	No specific or potentially actionable expression signatures
	Stroma-rich	15%	No specific or potentially actionable expression signatures
	Basal/squamous	35%	Express high levels of EGFR. Also express immune checkpoint markers, and may be more responsive to ICI
	Neuroendocrine-like	3%	No specific or potentially actionable expression signatures

Abbreviations: EGFR, epidermal growth factor receptor; FGFR, fibroblast growth factor receptor; HER2, human epidermal growth factor receptor-2; ICI, immune checkpoint inhibitor; UC, urothelial carcinoma.

## Biological Role of FGFR in the Pathogenesis of UC

**Figure 1.**
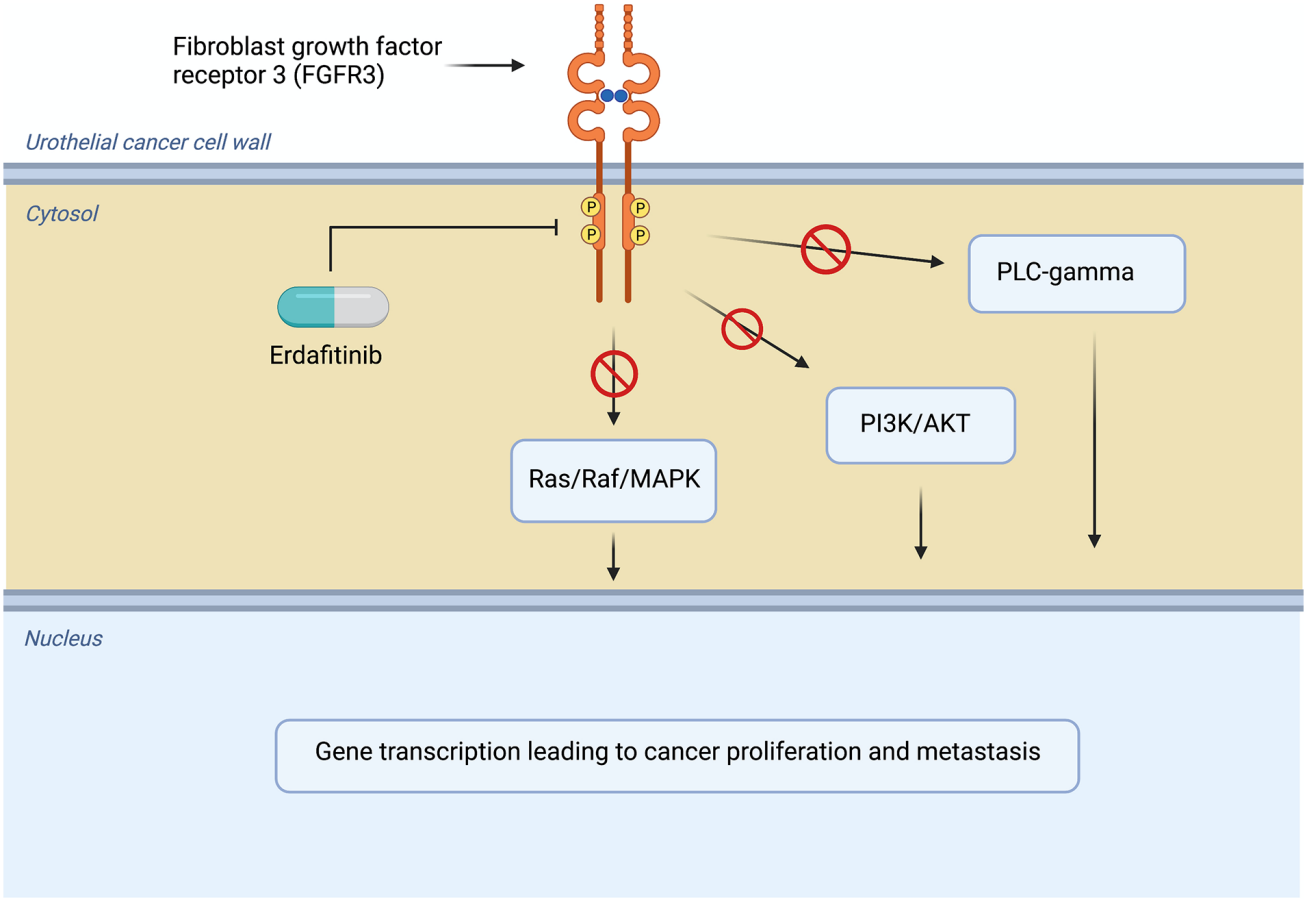
FGF/FGFR signaling pathway. AKT indicates Ak strain transforming; FGFR, fibroblast growth factor receptor; MAPK, mitogen-activated protein kinase; PI3K, phosphoinositide 3-kinase. Source: Adapted from BioRender.com.^17^ License permits BioRender content to be sublicensed for use in *Clinical Medicine Insights: Oncology*.

Fibroblast growth factor receptor alterations are common in up to 80% of stage Ta tumors. However, in stage T1 tumors and MIBC, FGFR3 mutations are less common and account for only 10% to 20% in tumors of stage T2 or above.^13^ A prior 2007 study by Tomlinson et al^14^ implicated FGFR3 in contributing to the risk of bladder cancer development. The FGR/FGFR signaling pathway plays a crucial role in several normal physiologic processes. Fibroblast growth factor receptors 1-4 are transmembrane receptors with tyrosine kinase domains. Following FGF ligand binding and FGFR receptor dimerization, the kinase domains trans-phosphorylate and lead to the docking of adapter proteins and the downstream activation of several key pathways.^15,16^ Ultimately, FGFR activation causes several changes including in proliferation, migration, and apoptosis with effects on intracellular signaling networks including phosphoinositide 3-kinase (PI3K)/ Ak strain transforming (AKT)/ mammalian target of rapamycin (mTOR) pathway, as well as mitogen-activated protein kinase (MAPK)/ extracellular-signal-regulated kinase (ERK) pathway,^16^ as shown in Figure 1.

The most commonly observed FGFR3 mutations in bladder cancer include S249C, Y373C, R248C, and G370C mutations.^18^ These mutations can occur either on the extracellular or transmembrane domains of the receptor, and ultimately lead to ligand-independent dimerization and activation. Regarding fusion proteins, FGFR: TACC3 fusions appear to have the highest occurrence in bladder cancer.^19^ Similar to mutation effect on the receptor, these fusion proteins lead to ligand-independent dimerization and activation of FGFR.^20,21^

## Role of Erdafitinib in FGFR-Altered UC

Erdafitinib is a potent FGFR inhibitor that is an oral pan-FGFR^1-4^ inhibitor. It is taken up by lysosomes, which results in sustained intracellular release and may contribute to its long-lasting activity.^22^ The single-arm phase II BLC2001 trial evaluated erdafitinib in 99 patients with metastatic or surgically unresectable UC and specific FGFR mutations or fusions. Eligible patients included those with progression on 1 or more lines of prior systemic chemotherapy or within 12 months of neoadjuvant chemotherapy, or patients who were chemo-naïve (cisplatin ineligible). The primary end point of the study was overall response rate (ORR), with secondary end points being progression free survival (PFS), overall survival (OS), safety, predictive biomarker evaluation, and pharmacokinetics.^23^ The BLC2001 trial showed an ORR 40% (3% complete response and 37% partial response), with notable secondary end points including a median PFS of 5.5 months, and median OS of 13.8 months. Of note, among patients who had previously undergone treatment with immunotherapy, the response rate was 59%. Grade 3 or higher treatment-related adverse events were reported in 46% of patients, and include stomatitis, central serous retinopathy, and hyperphosphatemia. However, a recent study indicates that hyperphosphatemia may be a marker of tumor response with FGFR inhibitors rather than a treatment-related adverse event.^24^ Following the BLC2001 trial, in April 2019, the Food and Drug Administration granted accelerated approval to erdafitinib in the treatment of mUC with susceptible FGFR3 or FGFR2 alterations that had progressed during or following platinum-based chemotherapy.^25^

Outside of the BCL2001 trial, data presented at ESMO 2019 used matching adjusted indirect comparison (MAIC) method to evaluate the efficacy of erdafitinib compared with available second-line therapies, including pemb-rolizumab, atezolizumab, docetaxel, vinflunine, paclitaxel, and mixed-chemotherapy. Results of MAIC method comparison show improvements in ORR, OS, and PFS with erdafitinib.^26^ There are several ongoing studies evaluating erdafitinib in the setting of metastatic or locally advanced UC. Erdafitinib is currently being studied in phase III THOR trial of erdafitinib versus chemotherapy or pembrolizumab in patients with advanced UC (NCT03390504). In addition, the ongoing phase IB/II NORSE trial is currently evaluating erdafitinib plus the PD-1 inhibitor JNJ-63723283 (Cetrelimab) in metastatic or locally advanced UC (NCT03473743).

## ICIs in FGFR-Altered UC

Several trials have demonstrated that luminal 1 subtype has shown to have the lowest response rate to anti-PD-L1 inhibitors atezolizumab and nivolumab compared with other subtypes.^27,28^ A study by Santiago-Walker et al^29^ evaluating PD-L1 treatment outcomes in patients with and without FGFR-altered advanced UC revealed that median OS in FGFR+ patients was lower than FGFR– patients (3.1 vs 6.1 months; hazard ratio [HR], 1.33; 95% CI, 0.78-2.26, *P* = .30) including on bivariate analysis. This study also suggested that FGFR+ status was associated with poorer OS in patients with any line of anti-PDL1 therapy (HR, 1.25; 95% CI, 0.71-2.21, *P* = 0.43). In addition, a retrospective study evaluating anti-PDL1 therapy in FGFR+ and FGFR– patients showed a trend toward lower ORR and disease control rate in FGFR+ patients in comparison with FGFR– patients who had received anti-PDL1 therapy. Multivariate analysis revealed a trend toward inferior OS in FGFR+ patients treated with immunotherapy (IO).^30^ Studies have proposed a lack of immune cell infiltration and immune marker expression as possible causes for the apparent lack of effectiveness of ICI in FGFR+ UC. Although PD-L1 status generally correlates with tumor response to ICI, it has been shown that there is no correlation between PD-L1 expression and ICI response in the treatment of non-small-cell lung cancer with a targetable mutation. In fact, it has been proposed that high PD-L1 expression in these cases may represent constitutive activation of the PD-L1 signal rather than as a marker of ICI responsiveness.^31^ It is possible that mUC with FGFR+ mutations and fusions may act similarly, and thus explain the lack of effectiveness of ICI.

## Future Perspectives

As discussed, the ongoing phase IB/II NORSE trial is currently evaluating erdafitinib plus the PD-1 inhibitor JNJ-63723283 (Cetrelimab) in metastatic or locally advanced UC (NCT03473743). Cetrelimab has demonstrated enhanced T-cell function and reversal of PD-1 mediated T-cell receptor signaling suppression based off in vitro assays.^32^ As previously discussed, luminal 1 tumors are reported to be enriched for FGFR3 mutations. However, these tumors lack in immune cell infiltration and immune marker expression.^33^ Not surprisingly, luminal 1 subtype has shown to have the lowest response rate to anti-PD-L1 inhibitors atezolizumab and nivolumab compared with other subtypes.^27,28^

**Figure 2.**
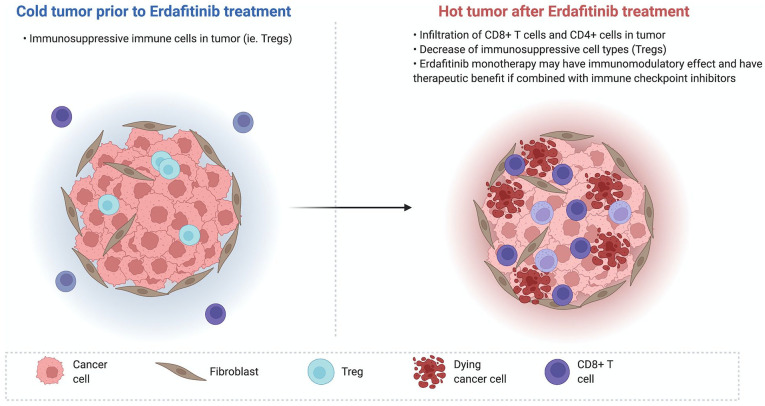
Immunomodulatory effect of erdafitinib. Source: Adapted from BioRender.com.^35^ License permits BioRender content to be sublicensed for use in *Clinical Medicine Insights: Oncology*.

Erdafitinib was studied alone and in combination with an anti-PD-1 therapy in a genetically engineered mouse model with lung cancer harboring mutations in FGFR and p53.^34^ The study showed that treatment with erdafitinib plus ICI showed statistically significant improvement in OS (median 19.7 weeks compared with 13.4 weeks, *P* < .0005 for combination vs control, and *P* < .004 for combination vs erdafitinib alone, log rank test). In addition, treatment with erdafitinib was shown to lead to infiltration of CD4+ and CD8+ T cells in the tumor, as well as decrease in the number of Tregs in tumors as shown in Figure 2. Therefore, this study demonstrated erdafitinib monotherapy may have an immunomodulatory effect, and have a potential therapeutic benefit when combined with ICI.

## Conclusions

The approval of ICI for the treatment has revolutionized the treatment of metastatic/advanced UC. More recently, the accelerated approval of the pan-FGFR inhibitor in mUC with susceptible FGFR3 and FGFR2 alterations has altered the treatment of FGFR+ UC. Although multiple prior studies have suggested a lack of effectiveness of ICI in FGFR+ UC, studies have demonstrated the immunomodulatory effect of erdafitinib in the tumor microenvironment. The ongoing NORSE trial of erdafitinib plus ICI offers the possibility of combined benefit from targeted therapy with ICI for patients with metastatic/advanced UC.
